# Targeting kynurenine metabolism in aging and age-associated disease

**DOI:** 10.1093/geroni/igaf122.1852

**Published:** 2025-12-31

**Authors:** George Sutphin

**Affiliations:** University of Arizona, Tucson, Arizona, United States

## Abstract

Tryptophan metabolism through the kynurenine pathway becomes dysregulated during normal aging and is implicated in many age-associated diseases, including chronic inflammation, atherosclerosis, neurodegeneration, and cancer. Kynurenine pathway enzymes and metabolites influence a range of molecular processes critical to healthy aging, including regulation of inflammatory and immune responses. Kynurenine metabolism is active in immune cells and activated in response to proinflammatory cytokine signaling. We discovered that elevating physiological levels of the kynurenine pathway metabolite 3-hydroxyanthranilic acid (3HAA) via either direct supplementation or inhibition of the enzyme that degrades 3HAA, 3HAA dioxygenase (HAAO), extends lifespan and delays age-associated health decline in both roundworms and mice. In recent work, we find that elevating physiological 3HAA can beneficially enhance both stress resilience and the immune response of C. elegans to bacterial pathogens during aging. In the absence of HAAO, 3HAA accumulates in lysosome related organelles (LROs) in the intestinal cells of C. elegans, the same subcellular compartment that contains engulfed bacteria. LROs are a cellular repository for both iron and zinc, and we further find that iron chelation or zinc supplementation dramatically enhances the bactericidal properties of 3HAA. Here we present a mechanistic model in which age-dependent accumulation of 3HAA in intestinal LROs combines with changes to iron and zinc homeostasis to enhance stress and bacterial pathogen resistance with age in C. elegans.

